# *Ganoderma lucidum* Polysaccharides Prevent Palmitic Acid-Evoked Apoptosis and Autophagy in Intestinal Porcine Epithelial Cell Line via Restoration of Mitochondrial Function and Regulation of MAPK and AMPK/Akt/mTOR Signaling Pathway

**DOI:** 10.3390/ijms20030478

**Published:** 2019-01-23

**Authors:** Zengenni Liang, Zhihang Yuan, Jiajing Guo, Jing Wu, Jine Yi, Jing Deng, Yang Shan

**Affiliations:** 1 Hunan Agricultural Product Processing Institute, Hunan Academy of Agricultural Sciences, Changsha 410125, China; enni_007@163.com (Z.L.); guojiajing2018@163.com (J.G.); 2 College of Veterinary Medicine, Hunan Agricultural University, Changsha 410128, China; zhyuan2016@hunau.edu.cn (Z.Y.); wujing@stu.hunau.edu.cn (J.W.); yijine@stu.hunau.edu.cn (J.Y.); 3 Hunan Academy of Agricultural Sciences, Changsha 410125, China; dengjing2020@163.com

**Keywords:** *Ganoderma lucidum*, polysaccharides, apoptosis, autophagy, mitochondria

## Abstract

*Ganoderma lucidum* polysaccharide (GLP) extracted from *Ganoderma lucidum* (Leyss. ex Fr.) Karst, a traditional Chinese medicine, is a biologically active substance reported to possess anti-oxidative, anti-apoptotic, and neurological protection. However, it is unknown whether GLP have any protective effect against high-fat constituents-induced epithelial cell injury. The aim of this study was to investigate the protection and molecular mechanism of GLP on injury induced by palmitic acid (PA) in the intestinal porcine epithelial cell line (IPEC-J2). First, we tested whether the treatment of GLP attenuate PA-induced IPEC-J2 cell death. GLP markedly blocked PA-caused cytotoxicity and apoptosis in IPEC-J2 cells. Moreover, GLP recovered the decreased mitochondrial function and inhibited activation of caspase-dependent apoptotic pathway. Interestingly, PA promoted cell apoptosis and autophagy through stimulation of phosphorylation of mitogen-activated protein kinases (MAPKs), AMP-activated protein kinase (AMPK), and inhibition of phosphorylation of Akt and mammalian target of rapamycin (mTOR), which was reversed by GLP. Taken together, this study revealed a protective effect of GLP against PA-evoked IPEC-J2 cell death through anti-apoptotic and anti-autophagic properties.

## 1. Introduction

The intestinal tract is an important organ that controls nutrient digestion and absorption. Nevertheless, it is extremely sensitive to high-fat constituents, and may be impaired by excess intake of high-fat foods though functional and morphological alterations related to intestinal permeability, which lead to malnutrition and inflammatory and metabolic disease [1]. Intestinal epithelial cells (IECs) are firmly organized into a single continuous layer and form the surfaces of the intestine to defend against foreign materials [2]. IECs can respond to a variety of potential risks to maintain organic homeostasis on a continuous basis in the mammalian gastrointestinal tract [3]. Hence, IECs are sentinels in intestinal homeostasis that facilitate the development of new approaches to protect against intestinal diseases [4].

It is becoming increasingly recognized that diet quality plays a crucial role in promoting intestinal health. Notably, a high-fat diet (HFD) is an important source of saturated fatty acids (e.g., lard and palmitic acid (PA)) that was found to be a statistically significant risk factor for development of diseases such as obesity, diabetes, angiocardiopathy, hyperproliferation, and cancer [5,6]. Mice exhibited the development of gross intestinal lesions and neoplasia that were histologically categorized as hyperplasia of intestinal mucosa and dysplastic crypts when maintained on the high-fat western-style diet for a duration of twenty-four months without any chemical intervention [7]. These mice also showed a significant enhancement of the short-term mitotic activity in the basal and intermediate portions of the colonic crypts, which simultaneously increased apoptosis of colonic epithelial cells [7].

*G. lucidum*, a Basidiomycetes mushroom, belongs to the family of Polyporaceae (or Ganodermataceae) of Aphyllophorals that grows in the tropical and subtropical areas of the word. Polysaccharides are one of the main bioactive components of *Ganoderma lucidum* linked by long-chain sugar molecules and glycosidic bonds. Clinical trials and other experimental studies indicated that *Ganoderma lucidum* polysaccharide (GLP) are responsible for several biological effects including anti-oxidative, antitumor, and neurological protection, and reportedly exerted significant effects on suppressing obesity and diabetes development [8,9]. Intraperitoneal injection of doses of GLP (50 and 100 mg/kg/d) in diabetic mice reduced epididymal fat/body weight ratio and fasting serum glucose levels, which related to low hepatic mRNA expressions of glycogen phosphorylase (GP) and glucose-6-phosphatase (G6Pase) and high mRNA levels of fatty acid synthase, acetyl-CoA carboxylase, and resistin in epididymal fat tissue [10,11]. This evidence indicated that GLP are potentially promising agents for obesity and diabetes therapy. However, to our knowledge, the roles of GLP in modulating high-fat constituents-mediated cell death in the intestinal tract have been poorly understood. Here, we intend to investigate the potential anti-cytotoxicity, anti-apoptotic, and anti-autophagic effects of GLP on PA-induced IPEC-J2 cells and to elucidate in detail the mechanisms underlying signaling pathways responsible for the anti-apoptotic and anti-autophagic role of GLP.

## 2. Results

### 2.1. GLP Suppressed PA-Mediated Cell Viability Loss in IPEC-J2 Cells

**Figure 1:**
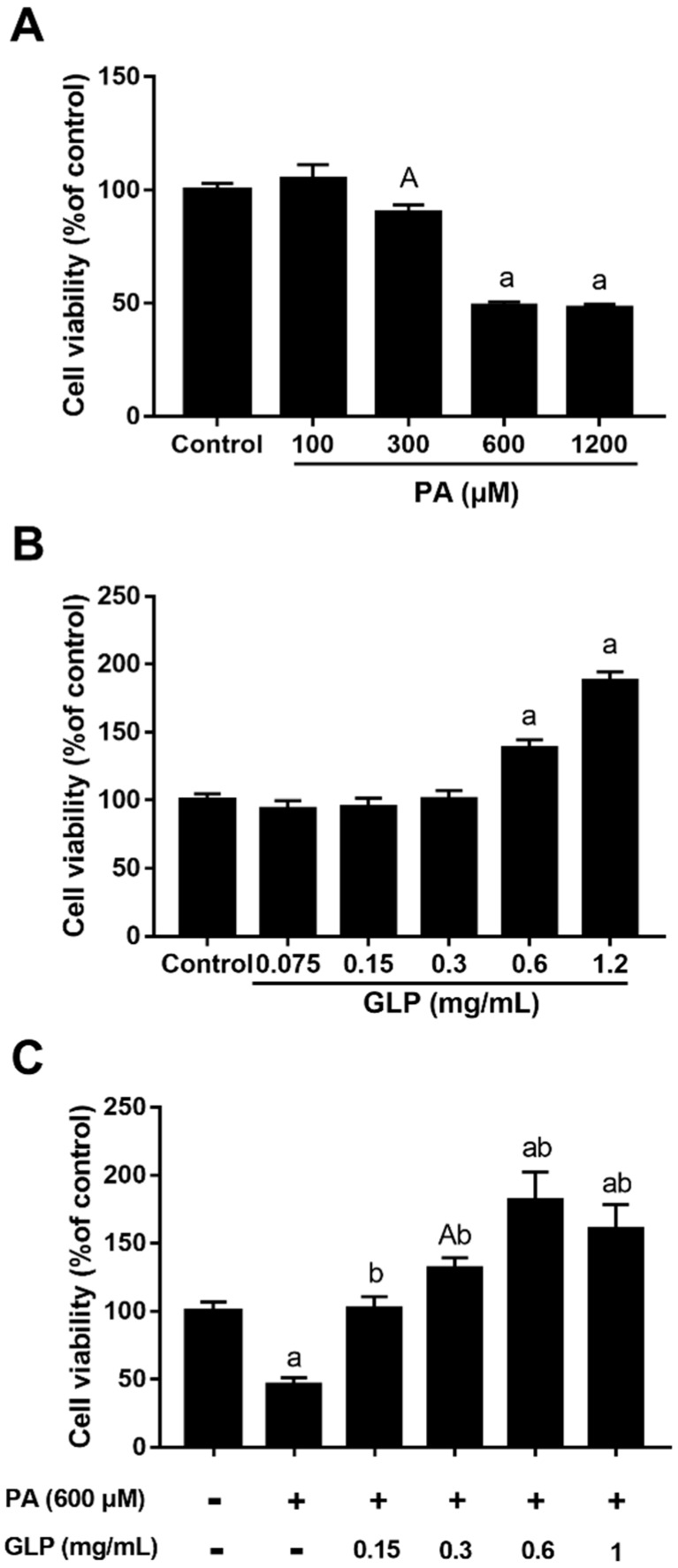
MTT assay determined the effects of palmitic acid (PA) and *Ganoderma lucidum* polysaccharide (GLP) on IPEC-J2 cell viability. Cells were treated with a 1640 medium containing 10% FBS (control), various concentrations of PA or/and GLP for 24 h. (**A**) Dose-dependent inhibitory effect of PA on IPEC-J2 cell viability. (**B**) The effect of various concentrations GLP (0.075–1.2 mg/mL) on IPEC-J2 cell viability. (**C**) The protective effect of GLP on PA-induced cell viability loss. Values are expressed as percentages of control and are as mean ± SE for three independent experiments (*n* = 5). A < 0.05 and a < 0.01 vs. control, b < 0.01 vs. PA alone.

When cells were treated with 100, 300, 600, and 1200 μM PA for 24 h, the inhibitory rate of cell viability was 0, 9.8%, 50.9% and 52.0%, respectively, and its IC50 value was 362.8 μM (Figure 1A). Since a 24 h incubation with PA reduced more than 50% of cell vitality at a concentration of 600 μM compared with control, we chose this concentration for subsequent assessments. In order to evaluate the toxicity of GLP, various concentrations of GLP (0–1.2 mg/mL) were incubated with cells for 24 h, and the cell viability was assayed by MTT. As shown in Figure 1B, treatment of GLP up to 1.2 mg/mL did not appear to have a negative effect on IPEC-J2 cell viability, suggesting no toxicity at these concentrations to the cells. In particular, high concentrations of GLP (0.6 and 1.2 mg/mL) resulted in an obvious increase in cell viability amounting to 139.0% and 188.0% of the control group, respectively. The potential protective effect of GLP was also determined in PA-induced IPEC-J2 cells. Figure 1C showed that GLP led to a dose-dependent inhibition of PA-induced cell viability loss (*p* < 0.01). In the presence of PA, high doses of GLP (0.3–1 mg/mL) stimulated markedly higher cell viability than control in IPEC-J2 cells.

### 2.2. Effect of GLP on Cell Morphology in PA-Induced IPEC-J2 Cells

**Figure 2:**
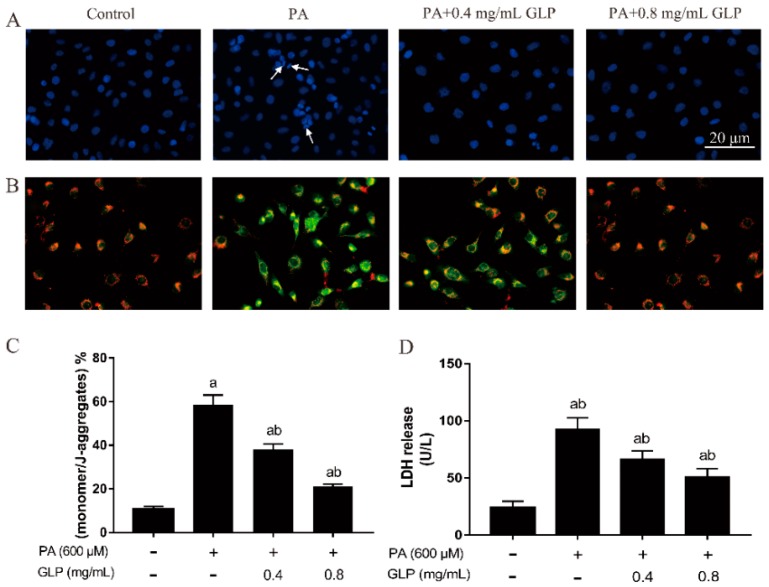
The effects of GLP on apoptotic characteristics in PA-induced IPEC-J2 cells. Cells were exposed to 600 μM PA with or without 0.4 and 0.8 mg/mL of GLP for 24 h. (**A**) Representative images of 4’,6-diamidi-no-2-phenylindole (DAPI) staining (blue). Arrows denote chromatin condensation and fragmentation. Original magnification 400×. (**B**) The mitochondrial membrane potential was examined by JC-1 staining. JC-1 aggregate and JC-1 monomer exhibited red and green fluorescence, respectively (400×). (**C**) JC-1 staining quantification results are presented as the mean of monomer/J-aggregates ± SE. (**D**) The release of LDH was analyzed using a commercially available kit and calculated as U/L. Data were presented as mean ± SE for three independent experiments (*n* = 3), a < 0.01 vs. control, b < 0.01 vs. PA alone.

4’,6-diamidi-no-2-phenylindole (DAPI) preferentially stains double-stranded DNA (dsDNA) in the nucleus. Consequently, it was usually used to assess cells with typical apoptotic characteristics [12]. As shown in Figure 2A, nuclei of untreated cells with blue fluorescence exhibited intact spherical structures and chromatin homogenously distributed in the nuclei. After cell treatment with 600 μM PA for 24 h, a lot of segmented nuclei with significant nuclear shrinkage, chromatin condensation, and fragmentation were observed in cells, as was evidenced by the appearance of prominent blue-colored semilune in PA-induced cells. On GLP treatment, most of cells displayed a spheric shape and uniformly stained chromatin, and the number of cells with chromatin condensation/fragmentation was lower in comparison to PA-treated cells. These results suggest that PA caused cell death by induction of apoptosis while GLP decreased PA-mediated apoptosis in IPEC-J2 cells.

### 2.3. GLP Restored PA-Caused Loss of Mitochondrial Membrane Potential in IPEC-J2 Cells

As shown in Figure 2B,C, untreated cells stained with JC-1 indicated red fluorescence, showing a high mitochondrial membrane potential. Exposure to 600 μM PA caused a color change in intracellular fluorescence from red to green in almost cells, suggesting that Δψm depolarization led to the monomer concentration reaching a high level. Similar to the effects obtained on MTT assay, cells incubated with GLP in presence of PA exhibited less green fluorescence and more red fluorescence in contrast to the PA-treated cells in dose-dependent manner. Furthermore, cells treated by positive control carbonyl cyanide 3-chlorophenylhydrazone (CCCP) exhibited an intense green fluorescence, indicating that a high depolarization of the mitochondrial membrane has occurred. These results suggest that GLP was capable of attenuating Δψm loss and suppressing the mitochondrial response.

### 2.4. GLP Alleviated the Increase of LDH Release in PA-Stimulated IPEC-J2 Cells 

LDH is a hydrogen transfer enzyme, which is used to measure the loss of cellular membrane integrity. To assess the effect of PA and GLP on cellular membrane integrity, LDH activity in cultured cell supernatant was evaluated after cells were incubated with GLP and PA for 24 h. Figure 2D showed the LDH release in untreated, PA-injured, and GLP-protected cells. Cellular LDH release significantly increased to 2.9-fold on 24 h of 600 μM PA incubation compared with that in untreated cells (*p* < 0.01; *n* = 5), indicating a significant cytotoxic effect on cellular membrane integrity induced by PA, whereas GLP and PA co-treatment could dose-dependently decrease an increase of LDH release. 

### 2.5. GLP Moderated PA-Triggered Apoptosis in IPEC-J2 Cells

**Figure 3:**
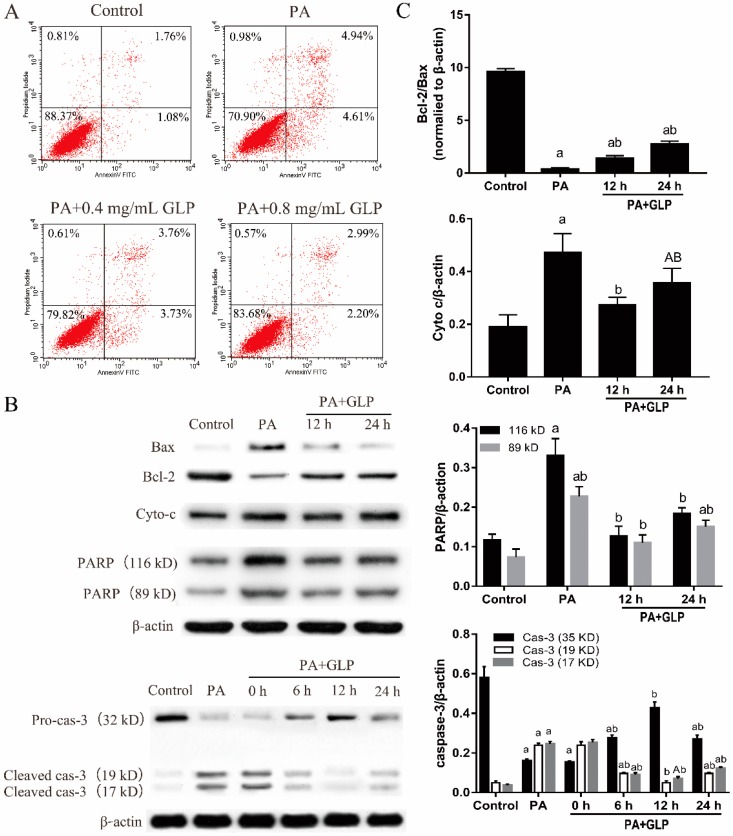
GLP prevented PA-induced apoptosis in IPEC-J2 cells. Cells were exposed to 0.4 and 0.8 mg/mL GLP in presence of 600 μM PA for 24 h. (**A**) Quantification of IPEC-J2 apoptotic cells by flow cytometry. Four parts were observed: necrotic cells (upper left quadrants), late apoptotic and necrotic cells (upper right quadrants), viable cells (lower left quadrants), and early apoptotic cells (lower right quadrant). (**B**) The expressions of Bax, Bcl-2, Cyto c, caspase-3 (cas-3), and PARP were analyzed by Western blot. (**C**) The data were quantified and normalized to β-actin. Data shown are representative of three independent experiments (*n* = 3). A < 0.05 and a < 0.01 vs. control, B < 0.05 and b < 0.01 vs. PA alone.

Initially, we performed flow cytometric assay in order to determine whether GLP alleviate PA-induced apoptosis in the target cells. As seen in Figure 3A, 600 μM PA caused an almost 7-fold increase in the percentage of apoptotic cells (early and late apoptotic cells) as compared with the controls (control, 3.07% ± 0.14%; PA, 11.81% ± 0.25%; *n* = 3). In contrast, the elevated apoptosis in PA-stressed IPEC-J2 cells was obviously decreased by 32.30% and 48.06%, respectively, in presence of both GLP and PA (*p* < 0.01). 

The potential molecular mechanisms underlying this protective effect of GLP on PA-induced cell apoptosis were investigated through measuring the expression of apoptosis-regulating proteins. As shown in Figure 3B,C, PA stimulated the expressions of pro-apoptotic protein Bax, Cyto c, and PARP, but reduced the expression of anti-apoptotic protein Bcl-2 compared with control (*p* < 0.01). Treatment with GLP for 12 and 24 h significantly prevented PA-induced cell apoptosis by upregulating Bcl-2 and downregulating Bax, Cyto c, and PARP (*p* < 0.05 or *p* < 0.01). Moreover, PA significantly increased caspase-3 activation, which was verified by the cleavage of caspase-3 and appearance of 17 and 19 kD active fragments, whereas GLP suppressed caspase-3 activation at 6, 12, and 24 h (*p* < 0.01). In PA-treated IPEC-J2 cells, the protein expressions of Cyto c, PARP, and caspase-3 activation were effectively inhibited by GLP exposure for 12 h. However, after cells were incubated with GLP in the presence of PA for 24 h, the rescuing effects of GLP on apoptosis-regulating proteins were weakened. Collectively, these findings validate that GLP can prevent PA-mediated apoptosis through the inhibition of mitochondrial pathway in IPEC-J2 cells.

### 2.6. GLP Modulated PA-Induced Energy Metabolism in IPEC-J2 Cells

**Figure 4:**
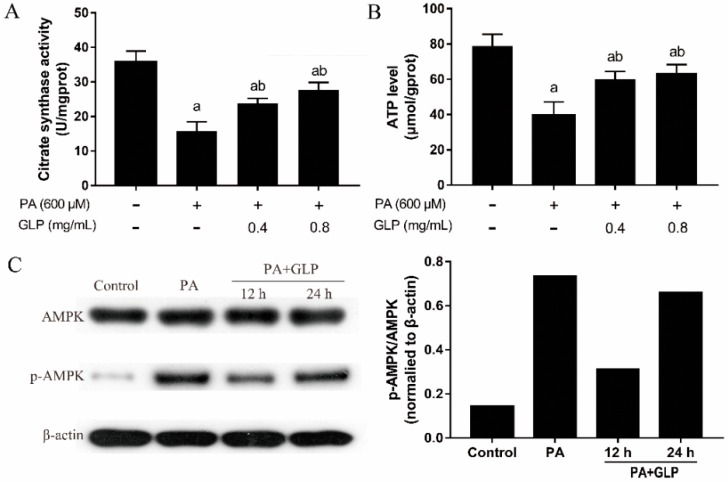
The effects GLP and PA on mitochondrial energy metabolism in IPEC-J2 cells. Cells were treated with 600 μM PA or/and GLP (0.4 and 0.8 mg/mL) for 24 h. (**A**) Citrate synthase activity was assayed using a citrate synthase assay kit and expressed as U/mg protein (*n* = 5). (**B**) Mitochondrial proteins were extracted using a mitochondria isolation kit after cells were lysed. The ATP level was determined by bioluminescence measurement and expressed as μmol/g protein (*n* = 5). (**C**) Representative Western blot of AMPK and p-AMPK. The data were quantified and normalized to β-actin. Data were presented as mean ± SE for three independent experiments, a < 0.01 vs. control, b < 0.01 vs. PA alone.

To elucidate the effects of GLP on PA-induced mitochondrial energy metabolism, CS activity, ATP content, and the levels of AMPK and p-AMPK proteins were measured. CS is a validated biomarker for cellular mitochondrial content associated with mitochondrial function [13]. As shown in Figure 4A, CS activity in PA-induced cells was 55.61% lower than that of control cells, whereas GLP (0.4 and 0.8 mg/mL) greatly rescued PA-reduced CS activity (*p* < 0.01; *n* = 5), suggesting that GLP can maintain cellular mitochondrial content and function in PA-induced cells. Change of mitochondrial ATP production plays a pivotal role in regulating apoptosis [14]. As shown in Figure 4B, PA-mediated cells exhibited a decrease of the level of ATP production (*p* < 0.01; *n* = 5) compared with the control cells, whereas GLP treatment greatly restored the decreased ATP generation in PA-induced cells. To understand whether cellular ATP depletion affects the activation of AMPK, the protein levels of AMPK and p-AMPK were detected by Western blot analysis. As seen in Figure 4C, PA stimulation markedly increased the protein level of p-AMPK compared with control. In contrast, GLP exposure markedly prevented AMPK activation by suppressing the phosphorylation state of AMPK (*p* < 0.01; *n* = 3). The phosphorylation of AMPK can be decreased at 12 h and increased with exposure time up to 24 h in cells incubated with PA in present of GLP.

### 2.7. GLP Inhibited PA-Induced Autophagy in IPEC-J2 Cells

**Figure 5:**
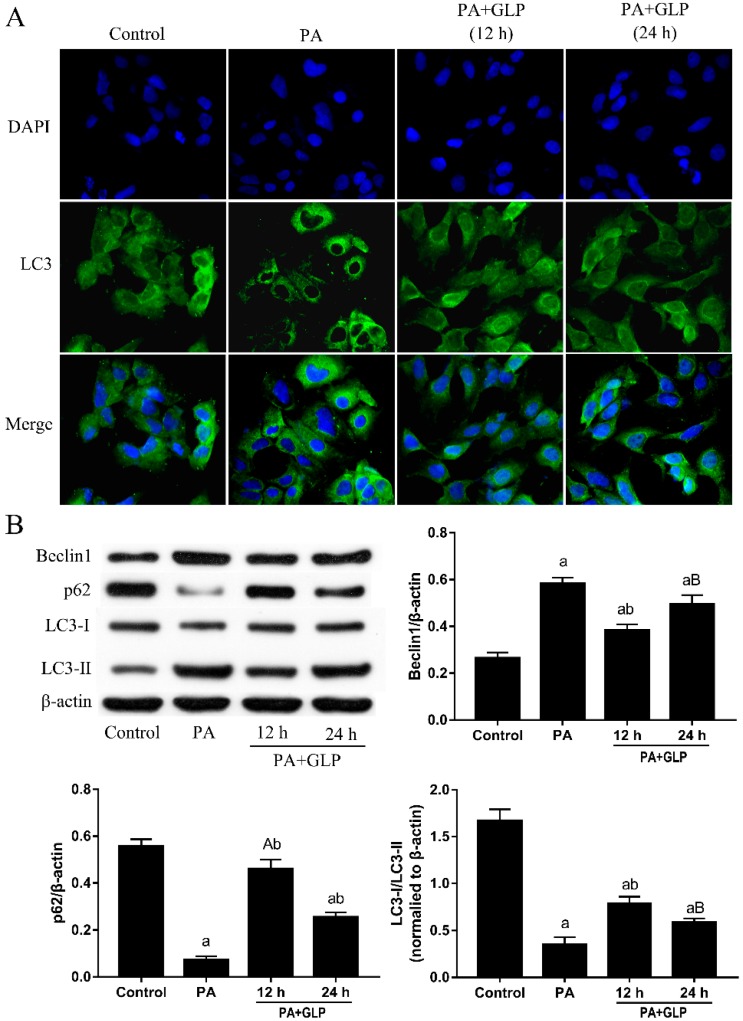
GLP ameliorated PA-induced autophagy in IPEC-J2 cells. Cells were treated with 600 μM PA and GLP (0.4 and 0.8 mg/mL) for 24 h. (**A**) Representative LC3/DAPI images. Cells were stained for LC3 (green) and DAPI (blue), and the fluorescence was visualized in a fluorescence microscope. Original magnification 400×. (**B**) The protein expressions of Beclin1, p62, LC3-I and LC3-II were subjected to Western blot analysis using indicating antibodies. The data were quantified and normalized to β-actin. Data shown are representative of three independent experiments (*n* = 3). A < 0.05 and a < 0.01 vs. control, B < 0.05 and b < 0.01 vs. PA alone.

We assessed whether GLP protect PA-induced cell death was involved in the anti-autophagic effect through endogenous LC3 immunofluorescence. As shown in Figure 5A, green fluorescence was diffused in the cytoplasm and the immunoreactivity of LC3 was weak in control cells. Compared to control cells, punctuated structures with bright green fluorescence were gathered on the autophagy membrane in cells treated with 600 μM PA for 24 h, whereas punctuated structures significantly less in cells exposed to PA plus GLP, indicating that PA-caused autophagy was inhibited by GLP in IPEC-J2 cells. We then further examined three autophagy-related proteins, namely LC3, p62 and Beclin 1. As shown in Figure 5B, the induction of autophagy in PA-treated IPEC-J2 cells was confirmed by an increase in Beclin 1 and LC3-II expressions, as well as a decrease of p62 and LC3-I expressions. However, autophagy inhibition with GLP markedly up-regulated p62, down-regulated Beclin 1 and blocked LC3 conversion from LC3-I to LC3-II in presence of PA. 

### 2.8. GLP Decreased the Phosphorylation of MAPK Signaling Pathway in PA-Induced IPEC-J2 Cells

**Figure 6:**
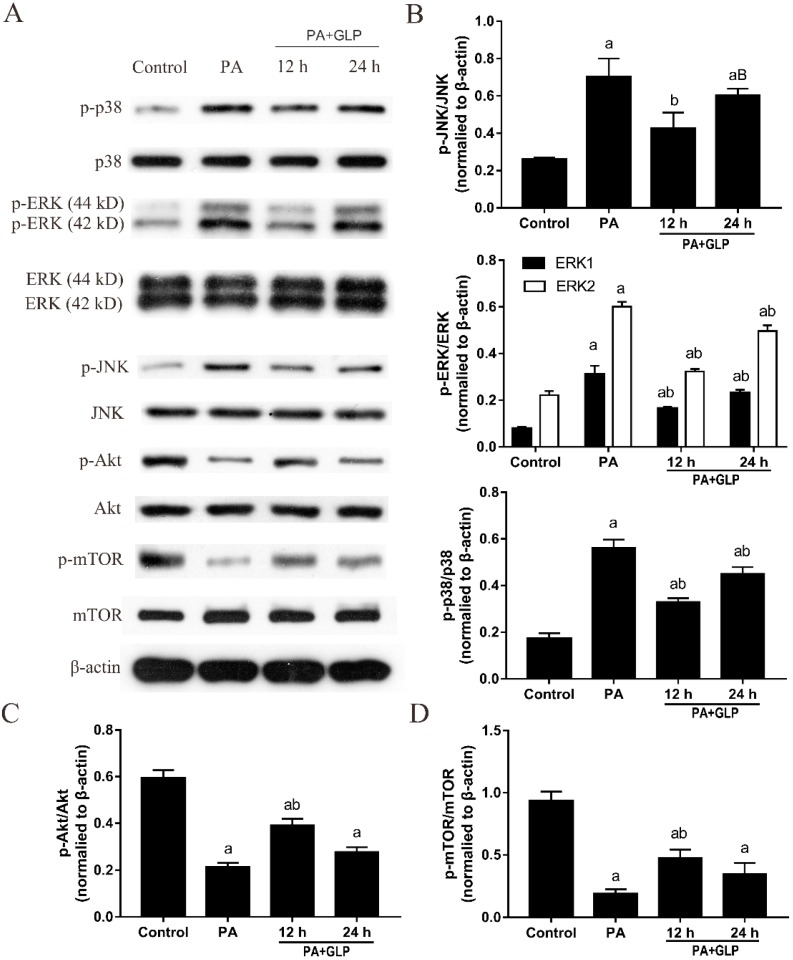
GLP activated Akt/mTOR signaling pathway and suppressed MAPK pathway in PA-induced IPEC-J2 cells. (**A**) Western blotting was used to analyze the protein expressions. (**B**) p-JNK/JNK, p-ERK/ERK, p-p38/p38, (**C**) p-Akt/Akt, and (**D**) p-mTOR/mTOR were calculated and presented. The data were quantified and normalized to β-actin. Data were presented as mean ± SE for three independent experiments (*n* = 3), a < 0.01 vs. control, B < 0.05 and b < 0.01 vs. PA alone.

To further determine the protective mechanism of GLP, total and phosphorylated MAPK pathway proteins were determined by Western blot. Results from Figure 6A,B showed that PA treatments resulted in significantly increased phosphorylation of JNK, p38, and ERK. Interestingly, we did not find any obvious influence on the expressions of JNK and p38 in any of treatment groups, although ERK, p-ERK, p-JNK, and p-p38 protein levels showed a significant change as compared with untreated cells. Compared with PA-untreated cells, GLP inhibited phosphorylation of MAPK signaling molecules, including p-ERK, p-JNK, and p-p38 with a maximum decrease at 12 h, indicating that GLP alleviate PA-stimulated cell death through inactivating MAPK signal transduction pathway.

### 2.9. GLP Suppressed Akt/mTOR Pathway Inactivation Mediated by PA in IPEC-J2 Cells

The Akt/mTOR signaling pathway is one of the most commonly altered pathways for regulating autophagy and apoptosis. Thus, we investigated whether this pathway is associated with PA-induced cell death, which also contributes to the protective effect of GLP. As shown in Figure 6A, PA decreased Akt and mTOR phosphorylation, whereas GLP partially increased Akt and mTOR phosphorylation without alteration in the expressions of Akt and mTOR. Additionally, compared with PA treatment, the ratios of p-Akt/Akt and p-mTOR/mTOR were significantly enhanced by GLP at 12 h (Figure 6C,D, *p* < 0.01), which was consistent with the peak of LC3-I/LC3-II ratio, indicating that Akt/mTOR signaling pathway may participate in the anti-autophagy and anti-apoptosis of GLP. 

## 3. Discussion

Phytochemicals have been used in pharmaceutical and dietary therapy including polysaccharides, alkaloids, steroids, carotenoids, phenolic glycosides, flavonoids, and other nitrogen-containing compounds [15]. The search for phytochemicals without toxic side effects that can protect IECs from damage is a crucial way toward that development of effective treatment strategies for intestinal diseases before overt clinical symptoms develop. Both pigs and humans are omnivores consuming plant and animal ingredients, who organs generally share common functional features [16]. In terms of genetics, the size and the composition of the porcine genome are comparable to humans. Moreover, pigs are very similar to humans in terms of anatomy and physiology. Highlighting these similarities, pig-to-primate xenotransplantation are being used successfully [17]. Therefore, the porcine cell model has numerous advantages for disease research. In our previous study, we found oral administration of GLP (200 and 400 mg/kg bw) for 12 weeks significantly decreased body weight, lipid accumulation, and the tissue index of the liver, heart, and white adipose tissues in mice fed on a HFD [18]. In this study, we developed an IPEC-J2 cell model to investigate the potential protection of GLP on intestinal epithelial cell death induced by high-fat constituent. The results unveiled that excessive PA cause impairment of cell viability in IPEC-J2 cells. However, PA-induced cell viability loss was prevented by GLP without cytotoxicity, which coincided with previous findings showing that GLP was no toxicity to normal cells [19].

Apoptosis, a cellular process for control of cell survival and death, participates in various physiological activities including cardioprotection and embryogenesis [20,21]. In the gastrointestinal tract, apoptosis contributes to maintain an appropriate number of cells by balancing newly generated and dead cells and to modulate cell number migrating up the crypt-villus axis [22]. Nevertheless, extensive apoptosis in IECs leads to severe intestinal pathology resulting in rapid weight loss and animal death [23]. Of note, numerous evidence has confirmed that mitochondria drive apoptosis by regulating expression of Bcl-2 family proteins, the release of Cyto c from mitochondria to cytoplasm, and activation of caspase-3 and PARP [24]. In accordance, we found that PA triggered a distinct apoptosis, which is connected to an increase of LDH release, caspase-3 cleavage, expressions of Bax, Cyto c, and PARP, as well as a decrease of Bcl-2 expression. However, GLP defended against PA-induced cell apoptosis via interruption of the mitochondrial apoptotic pathway. 

Mitochondria are also the principal site for biochemical energy production, as ATP synthesis is produced from the inner surface of the mitochondrial inner membrane in higher organisms (yeast, plants, and mammals). CS localized in mitochondria are a key important limiting enzyme of tricarboxylic acid cycle, and its activity is inhibited by the level of the sum of the energy-rich phosphate bonds of ATP and ADP. On the contrary, data from a recent study reveal that CS can govern intracellular ATP synthesis. For example, RNAi-mediated CS knockdown in human cervical carcinoma cells impaired Δψm, accelerated cytosolic glycolysis, and reduced ATP production [25]. AMPK is a key sensor of cellular energy status and regulates cellular metabolism to sustain energy homeostasis. Under conditions of low intracellular ATP, AMP binding stimulates AMPK by way of allosteric stimulation of phosphorylated AMPK and subsequently suppression of cell proliferation and biosynthetic processes and promotion of autophagy [26]. In the present study, PA exposure induced a decrease of ATP production and activation of AMPK, which may be partially responsive to the decreased CS activity, whereas GLP could effectively reverse the effect of PA on the energy metabolism. It was concluded that these critical alterations in mitochondrial energy metabolism contribute to the protective effect of GLP on mitochondrial function and biogenesis in PA-induced cells [27], a result similar to that presented by Dagon et al. [28].

Autophagy is a self-degradative cellular process that plays a wide variety of pathophysiological and physiological roles in removing intracellular pathogen, balancing sources of energy, eliminating abnormal aggregated proteins, clearing damaged and unwanted cells in normal multicellular organisms [29,30]. In mammalian cells, the cytoplasmic form LC3-I is processed and recruited to autophagosomes, and in turn lapidated to LC3-II by site-specific proteolysis, thereby inducing autophagy. The protein Beclin 1 is an essential mediator of autophagy, which BH3-like domain interacts with Bcl-2, and then regulates autophagosome formation and autophagy [31]. The major cargo receptor p62 affects the protein interaction between Bcl-2 and Beclin 1 through association with Bcl-2 and directly binds to the two autophagy effectors LC3-I and LC3-II during the autophagy process [32]. As reported, p62 aggregation in autophagy-deficient mice suggests a negative link between autophagy and p62, which could promote caspase-activated apoptosis [33]. It is noteworthy that AMPK triggers FoxO3a transcription factor activation, resulting in upregulation of the autophagy-associated proteins Beclin 1 and LC3-II in Tibialis anterior muscle and in primary mouse skeletal muscle myotubes [34]. In this study, the remarkably inhibitory effect of GLP on AMPK activation in PA-stimulated IPEC-J2 cells observed led us to think about whether these beneficial effects of GLP could be related to autophagy suppression. To investigate this possibility, autophagosomes were assessed using DAPI/LC3 staining and autophagy-related protein expressions were measured by Western blot analysis. The results provide support for the hypothesis that GLP exposure dampens PA-evoked autophagy in IPEC-J2 cells by the promotion of p62 expression level and reduction of autophagosome number, Beclin 1 protein expression and LC3-II/LC3-I conversion, which may be another important metabolic mechanism associated with the protection of GLP against IPEC-J2 cell death induced by PA. 

MAPKs including JNK, p38, and ERK play vital roles in regulating cell growth, differentiation, migration, senescence, and the process of apoptosis and autophagy [35]. Both JNK and p38 MAPKs synchronously stimulated by a variety of endogenous and exogenous stresses adjusted the balance of cell apoptosis and autophagy [36]. The activation of JNK and p38 triggers autophagy as a pro-survival mechanism for cells to against the apoptotic and cytotoxic effects [37]. ERK is a survival factor in a central position for the cellular decision, the function of which has been depicted as growth stimuli, in essence by combating the upregulation of pro-apoptotic genes activated by JNK/p38 MAPK pathways [38]. However, recent reports have observed contrary results correlated with previous findings on the role of ERK pathway in cell survival. Kim et al. has reported that salinomycin-induced apoptosis was associated with ROS-mediated autophagy in human prostate cancer cells through regulation of ERK/p38 MAPK signaling pathway, and the reaction was suppressed by pretreatment with an ERK inhibitor [39]. This study indicated that GLP treatment effectively alleviated PA-mediated enhancement of p38, JNK, and ERK phosphorylation. The inhibition of MAPK pathway activation may contribute to suppressing PA-induced apoptosis and autophagy in IPEC-J2 cells. The results are similar to the conclusion in the studies of Liu et al. and Wei et al. In H9c2 cardiomyoblast cells, PA was reported to induce cell autophagy and apoptosis by stimulating p38 and JNK MAPK pathways as well as promoting the conversion of LC3-I to LC3-II [40]. ERK1/2 inhibitor, U0126, reduced the activated ERK and PA-induced apoptosis in rat cardiac muscle H9c2 cells through partial inhibition of intracellular ROS production [41]. 

Akt activation induces cell cycle progression, migration, and metabolism, whose downstream target mTOR is well-known as an essential regulator of protein synthesis, cell growth, and proliferation in mammalian cells [42]. In particular, dysregulation of mTOR signaling contributes to cell autophagy and apoptosis by modulating various signal molecules, such as inhibition of Akt activity and promotion of AMPK [43]. Constitutive activation of the Akt/mTOR pathway increases cell survival and meanwhile prevents apoptosis and autophagy in an uncontrolled manner [44]. In this study, exposure of PA obviously decreased the Akt and mTOR phosphorylation levels in IPEC-J2 cells. However, GLP rescued PA-medicated reduction in p-Akt/Akt ratio and p-mTOR/mTOR ratio. Thus, another novel finding of our present study is that GLP can prevent IPEC-J2 cells from PA-induced cell death via modulation of Akt/mTOR pathway. Interestingly, some reports found that induction of Akt and mTOR expressions can regulate Bcl-2 phosphorylation. Suppression of mTOR signaling by the expression of a dominant negative mutant of the Akt kinase cause an increase in Bcl-2 phosphorylation in HEK23 human embryonic kidney cells [45], suggesting that Akt inactivation may inhibit mTOR expression and upregulate Bcl-2 phosphorylation, thereby preventing cell apoptosis. Nevertheless, different results were obtained in this study, that is, GLP suppressed cell autophagy and apoptosis caused by PA via inactivation of Akt/mTOR signaling pathway and accompanied by inhibition of Bcl-2 phosphorylation in IPEC-J2 cells. 

Results from the expression of target proteins including Cyto c, PARP, caspase-3, p-AMPK, Beclin 1, p62, LC3-I, LC3-II, p-p38, p-JNK, p-ERK, p-AKt, and p-mTOR in apoptotic and autophagic processes show that GLP displayed effectively anti-apoptotic and anti-autophagic effects at incubation 12 h in PA-incubated IPEC-J2 cells, whereas the strength of the rescuing effects of GLP were weakened at incubation 24 h, but still significant. The trend of results was similar with previous work those of Pan et al., whom reported that 100 μM Cordycepin induced cleavage of caspase-3, –6, –7, –8, and PARP with maximal cleavage at 12 h in mouse Leydig tumor cells [46]. Most drugs enter cells through diffusion or endocytosis, then are absorbed and metabolized in a variable fashion. Accordingly, the concentration of drugs becomes decreased in conditioned media of cells, and the drug effect gradually diminish. This may be the reason that the rescuing effects of GLP was progressively lost with the extension of incubation time.

## 4. Materials and Methods 

### 4.1. Preparation of GLP

GLP were isolated from the fruit bodies of *Ganoderma lucidum* as described by Liang et al. [47]. Structural characteristics of the extract were investigated using UV spectra, infrared spectra, and high-performance anion exchange chromatography (HPAEC). These findings suggested that the extract was composed of acidic polysaccharides containing 11% uronic acid and 89% total carbohydrate. GLP were mainly comprised of cellose, glucose, galactose, and arabinose in the mole percentages of 5.56%, 16.67%, 16.67%, and 61.11% with α-type glycosidic linkage. The molecular weight range were 10–30 kDa (32.1%), 30–50 kDa (21.8%), and >50 kDa (46.1%), respectively. Freeze-dried GLP powder was dissolved in RPMI 1640 media with 10% fetal bovine serum (FBS), filter sterilized, and stored at −20 °C for later analysis.

### 4.2. Cell Line and Culture

IPEC-J2 cell line isolated from a neonatal, unsuckled piglet and immortalized with the human telomerase reverse transcriptase (hTERT) gene [48,49] was obtained from Hunan Agricultural University. Cells were maintained in 1640 medium with 10% FBS at 37 °C under a humidified atmosphere containing 5% CO2. PA (Sigma, St. Louis, MO, USA) was dissolved in 100% ethanol at 200 mM and then diluted in 1640 medium containing 10% fatty acid-free bovine serum albumin (BSA, Sigma, St. Louis, MO, USA) to a concentration of 50 mM. The final concentration of ethanol in all experiments was lower than 0.5% *v/v* in cell culture media. The stock solutions were adjusted to pH 7.4, filtered through 0.22 μm filter membranes, and stored in refrigerator until ready for use. 

### 4.3. Assessment of Cell Viability by MTT

Cells (1 × 10^5^ cells/mL) were seeded in 96-well plates for 24 h and then treated with various concentrations of PA or/and GLP for 24 h. At the end of the treatment, cell viability was determined at 490 nm by an ELISA reader (Thermo Multiskan MK3, Waltham, MA, USA) using the colorimetric 3-(4,5-Dimethylthiazol-2-yl)-2,5-diphenyltetrazolium bromide (MTT, Beyotime Biochemical Co., Shanghai, China) according to the manufacturer’s instructions as described previously [50]. Cell viability (%) was calculated as [(sample OD value−reference OD value)/control OD value] × 100%. All the samples were conducted in five replicates.

### 4.4. DAPI Staining 

After cells (2 × 10^5^ cells/mL) were incubated with 600 µM PA or/and GLP (0.4 and 0.8 mg/mL) for 24 h, nuclear morphology was analyzed by DAPI staining. In brief, treated cells were centrifuged, washed with phosphate buffer saline (PBS, pH7.4) one time, and fixed in 4% paraformaldehyde for 0.5 h at room temperature. The samples were washed in PBS three times for 10 min each before staining with 1 μg/mL of DAPI (Sigma, St. Louis, MO, USA) in the dark at 37 °C for 30 min. Subsequently, each sample was washed with PBS six times to remove unbound DAPI and observed under a fluorescence inverted microscope (Olympus, CKX41SF, Tokyo, Japan).

### 4.5. Detection of Mitochondrial Membrane Potential

The mitochondrial membrane potential (Δψm) was examined with a Δψm-dependent lipophilic dye JC-1. 5,5’,6,6’-tetrachloro-1,1’,3,3’-tetraethylbenzimidazolylcarbocyanine iodide (JC-1, St. Louis, MO, USA) were dissolved in dimethyl sulfoxide (DMSO, St. Louis, MO, USA) and diluted in 1640 medium supplemented with 10% FBS. After treatment, cells were incubated with 1 mL of 5 μM JC-1 or CCCP (Sigma, St. Louis, MO, USA) for 20 min at 37 °C in the dark, washed with ice-cold PBS and added cellular medium. Finally, JC-1 aggregate and JC-1 monomer fluorescence was analyzed using an inverted fluorescence microscope (Olympus, CKX41SF, Tokyo, Japan) and a multifunctional microplate reader (TECAN, Infinite 200 PRO, Grodig, Austria) by excitation/emission at 525 nm/590 nm (red fluorescence) and 490 nm/530 nm (green fluorescence) wavelength, respectively. The mitochondrial potential disrupter CCCP was used as a positive control.

### 4.6. Cell Membrane Integrity Assay

IPEC-J2 cells (2 × 10^5^ cells/mL) were seeded in 96-well plates overnight followed by incubation with GLP (0.4 and 0.6 mg/mL) or/and 600 μM PA for 24 h. At the end of the incubation period, cells were centrifuged at 3000× *g* for 10 min, and then the cultured supernatant was transferred to new 96-well plates. To measure cell membrane integrity, LDH release was detected at 450 nm by a microplate reader (Molecular Devices, Sunnyvale, CA, USA) using a Lactate dehydrogenase (LDH) assay kit (Nanjing Jiancheng Bioengineering Institute, China). 

### 4.7. Apoptosis

Cells (2 × 10^5^ cells/mL) were exposed to 600 μM PA with or without GLP (0.4 and 0.8 mg/mL) for 24 h. After washed twice with pre-chilled 0.01 M PBS (pH 7.4), cells were suspended in 500 μL 1× binding buffer prior to exposure to 5 μL of Annexin V- Fluorescein isothyocyanate (FITC, BD Biosciences, San Jose, CA, USA) and 5 μL of propidium iodide (PI) for 15 min at room temperature in the dark. Cell apoptosis was monitored by flow cytometry (Beckman Coulter, Inc., Miami, FL, USA) within 1 h.

### 4.8. Citrate Synthase Activity Assay

Citrate synthase enzyme assay was performed spectrophotometrically using a citrate synthase assay kit (Nanjing Jiancheng Bioengineering Institute, China). After treatment, cells were lysed with ice-cold RIPA buffer (150 mmol NaCl, 0.1% Triton X-100, 0.5% sodium deoxycholate, 50 mmol Tris-HCl, pH 8.0), and the total protein concentrations were determined using a BCA kit (Beyotime Biochemical Co., Shanghai, China). The cell lysate was added with a reaction mixture consisting of 5 mM triethanolamine hydrochloride, 100 mM Tris-HCl (pH 8.0), 30 mM acetyl-CoA, 1 M EDTA, 10 mM 5,5’-dithiobis-(2-nitrobenzoic acid) (DTNB) and 10 mM oxaloacetate (OAA). The absorption was measured spectrophotometrically at 412 nm at 25 °C and expressed as U/mg protein.

### 4.9. Determination of the Level of Mitochondrial ATP

IPEC-J2 cells (2 × 10^5^ cells/mL) were incubated 600 μM PA or/and GLP (0.4 and 0.8 mg/mL) for 24 h. After cells were lysed with ice-cold RIPA buffer, mitochondrial proteins were extracted using a mitochondria isolation kit. Subsequently, protein concentrations were determined using a BCA protein assay kit. The mitochondrial ATP level was quantified using an ATP assay kit (Nanjing Jiancheng Bioengineering Institute, China) according to the manufacturer’s instructions, determined by bioluminescence measurement using a UV–Vis Shimadzu UV-1800 spectrophotometer (Shimadzu Corporation, Kyoto, Japan) at 560 nm and expressed as μmol/g protein.

### 4.10. Immunofluorescence

Cells (2 × 10^5^ cells/mL) were exposed to 600 μM PA with or without GLP (0.4 and 0.8 mg/mL) for 24 h. Treated and untreated cells were fixed by 4% paraformaldehyde, permeabilized with 0.3% Triton X-100 and blocked with 5% BSA in PBS. Subsequently, the samples were incubated with LC3B antibody (1:100) overnight at 4 °C. Following washes with PBS, the coverslips were incubated with 150 μL Alexa Fluor 488-conjugated donkey anti-rabbit IgG (H+L) for 1.5 h at 37 °C. Cell nuclei were counterstained with DAPI. Fluorescent images were examined using a fluorescence microscope (Olympus, CKX41SF, Tokyo, Japan). 

### 4.11. Western Blot Analysis

IPEC-J2 cells (2 × 10^5^ cells/mL) were seeded in six-well plates with 600 μM PA and different concentrations of GLP at corresponding time, and then total protein were extracted from IPEC-J2 cell pellet. Protein concentration was estimated using BCA assay and stored at −80 °C until analysis. Twenty microgram total protein were diluted in loading buffer, separated on 10% and 12% sodium dodecyl sulphate-polyacrylamide (SDS-PAGE) gels electrophoresis and transferred onto polyvinylidene fluoride (PVDF) membranes. The blots were blocked in Tris-buffered saline containing 0.1% Tween 20 (TBST) supplemented with 5% BSA, and then probed with the primary antibodies at 4 °C overnight. After three washes with TBST, the membranes were probed with HRP secondary antibodies (Proteintech, Chicago, IL, USA) at room temperature for 1 h. β-actin served as an internal control. The specific proteins were visualized using an enhanced chemiluminescence detection kit according to the manufacturer’s instructions. Densitometry was performed using ImageJ software (v.1.8).

### 4.12. Statistical Analysis

Results are presented as means ± standard error (SE). Statistical analysis was performed with Statistical Program for Social Sciences v.18.0 (SPSS Inc., Chicago, IL, USA) software. Multiple comparisons were made using an analysis of variance (ANOVA) for repeated measures followed by Tukey’s post hoc test. A significant difference was considered as *p* < 0.05 and an extremely significant difference was expressed as *p* < 0.01.

## 5. Conclusions

In summary, our study provides the first evidence that GLP could reverse PA-induced cytotoxicity by concomitantly inhibiting apoptosis and autophagy in IPEC-J2 cells without cytotoxicity. GLP effectively suppressed apoptosis and autophagy via restoration of mitochondrial function, inactivation of the mitochondrial, MAPK, and Akt/mTOR signaling pathways. Further studies are required to determine the precise mechanisms of cell death, as well as the interaction between signaling pathways related to apoptosis and autophagy in vitro.
